# Variability of the Ki-67 proliferation index in gastroenteropancreatic neuroendocrine neoplasms - a single-center retrospective study

**DOI:** 10.1186/s12902-018-0274-y

**Published:** 2018-07-28

**Authors:** Huiying Shi, Qin Zhang, Chaoqun Han, Ding Zhen, Rong Lin

**Affiliations:** 1 Department of Gastroenterology, Union Hospital, Tongji Medical College, Huazhong University of Science and Technology, Wuhan 430022, China; 2 Department of Pathology, Union Hospital, Tongji Medical College, Huazhong University of Science and Technology, Wuhan 430022, China

**Keywords:** Gastroenteropancreatic neuroendocrine neoplasms, Ki-67, Metastases, Variability, Prognostic factors

## Abstract

**Background:**

The Ki-67 index in gastroenteropancreatic neuroendocrine neoplasms (GEP-NENs) may change throughout the disease course. However, the definitive effect of Ki-67 variability on GEP-NENs remains unknown. The aims of this study were to evaluate changes in Ki-67 levels throughout the disease course and investigate the role of Ki-67 index variability in GEP-NENs.

**Methods:**

Specimens with multiple pathologies were evaluated from 30 patients who were selected from 514 patients with GEP-NENs, being treated at Wuhan Union Hospital from July 2009 to February 2018. The Ki-67 index was evaluated among multiple specimens over the disease course. Univariable and multivariable Cox proportional hazards regression analyses were performed to assess the prognostic significance of various clinical and histopathologic features.

**Results:**

Among the 514 patients with GEP-NENs, metastases were seen in 182 (35.41%). Among the 30 patients from whom specimens with multiple pathologies were obtained, 24 were both primary and metastatic specimens and six were specimens collected over the course of the disease. Changes in Ki-67 levels were detected in 53.3% of the patients, of whom 40% had up-regulated Ki-67 levels, and 13.3% had down-regulated Ki-67 levels. Kaplan–Meier survival analysis showed that the group with Ki-67 variability had a shorter overall survival (*p* = 0.0297). The Cox regression analysis indicated that Ki-67 variability (*p* = 0.038) was the only independent prognostic factor for overall survival.

**Conclusions:**

Our data suggest that patients with GEP-NENs and Ki-67 variability had a poorer prognosis. The re-assessment of Ki-67 at sites of metastasis or during the disease course might play a role in predicting the prognosis of patients with GEP-NENs. This finding could have implications for how GEP-NENs are monitored and treated.

## Background

The Ki-67 protein, a cell proliferation-associated nuclear marker, has become a useful tool in assessing the malignant potential of neuroendocrine neoplasms (NENs) [1–3]. With respect to gastroenteropancreatic neuroendocrine neoplasms (GEP-NENs), the Ki-67 labeling index had already become an integral part of the World Health Organization (WHO) classification, from as early as the 2004 edition [4]. Subsequently, in the WHO-2010 classification schemes, GEP-NENs were further subdivided into three grades by the Ki-67 index as follows: grade 1 (G1) tumors with a Ki-67 index ≤2%; (grade 2) G2 tumors with an index of 3–20%; and (grade 3) G3 tumors, > 20% [5]. For NENs, a higher Ki-67 labeling index is associated with a poorer prognosis [2, 6, 7]. Accordingly, the grading system based on Ki-67 facilitates identification of the aggressive subset of NENs and provides a standardized diagnostic pathway that is appropriate for effective decision-making in the management of NENs.

The GEP-NENs, one kind of heterogeneous tumors, frequently present with metastatic deposits at initial diagnosis; distant metastases have been suggested to be one of the strongest predictors of survival in GEP-NENs [1, 8–10]. In recent years, several publications have noted that the Ki-67 index varies from the site of the primary tumor to those of metastases, and even throughout the disease course [1, 2, 11]. In addition, researchers have also advocated that a sufficient evaluation of the Ki-67 index in metastatic tumors could have prognostic value and might be necessary to optimize clinical decision-making.

Despite the fact that discordant expression of Ki-67 exists at the primary and metastatic tumor sites, little is known about the manner in which Ki-67 variability changes throughout the disease, and thus affects prognosis. This is partly due to low prevalence and a lack of large sample data on GEP-NENs. We reviewed 514 patients with GEP-NENs and assessed the Ki-67 levels in the subgroup whose specimens bore multiple pathologies. Our aims were to explore the relationship between Ki-67 variability and prognosis in patients with GEP-NENs and provide the basis for more accurate decision-making in GEP-NENs.

## Methods

We conducted a single-center retrospective study. The present study retrospectively reviewed patients diagnosed with GEP-NENs at Wuhan Union Hospital from July 2009 to February 2018. A total of 514 patients were included in the study, among which, 30 had multiple specimens taken from the primary tumor and a metastatic focus, or during the course of the disease. The diagnosis of NENs was performed through conventional histological and immunohistochemical analysis of specimens from the primary tumor and/or metastatic lesions. All specimens obtained by surgical resection, fine needle aspiration, and/or core biopsy were made available for all enrolled patients. The medical records were retrospectively reviewed to collect the following data: age, sex, primary and metastatic tumor sites, and Ki-67 labeling index.

The study was approved by the Ethics Committee of Tongji Medical College, Huazhong University of Science and Technology (IORG No: IORG0003571), and performed in accordance with the Declaration of Helsinki. As it was a retrospective study, all data were collected from a medical records system. Therefore, the study was exempt from the requirement to obtain individual informed consent, based on the Ethical Guidelines of the Ethics Committee of Tongji Medical College, Huazhong University of Science and Technology.

### Immunohistochemistry

The specimens were fixed in 4% paraformaldehyde and embedded in paraffin wax. To evaluate the Ki-67 proliferation index of tumors, the paraffin-embedded tissue blocks were cut into 4 μm thick sections, and tissue sections were then assessed by immunohistochemistry with a Ki-67 antibody (MIB-1, DAKO), using the Ventana Discovery staining system (Ventana Medical Systems). The Ki-67 index was determined by calculating the percentage of tumor cells with positive staining, among up to 2000 tumor cells in the densest field of each slide. All results were verified by two pathologists (QZ and the pathologist responsible for the original pathology report).

### Statistical analysis

Clinical and pathologic characteristics of patients were expressed as median and range, or percentage. Overall survival was defined as that time from the date of diagnosis to the date of death, or last follow-up. Survival curves were drawn according to the Kaplan–Meier analysis, and differences between groups were assessed using the log-rank test. Cox proportional hazards regression analysis was used to assess prognostic factors for survival. Statistical calculations and data manipulation were performed using the SPSS software v21.0 (IBM, USA), and *p* < 0.05 was considered statistically significant.

## Results

### Patient characteristics

Among the 514 patients with a GEP-NENS diagnosis, 302 (58.75%) were men and 212 were (41.25%) women. The median age at the time of diagnosis was 55 years (range: 12–85 years). Of the 514 GEP-NENs patients, 196 (38.13%) cases were of low grade G1; 102 (19.84%) of intermediate grade G2; and 216 (42.02%) of high grade G3. Metastases were observed in 35.41% (182/514) of all cases, and in 9.18% (18/196) of G1 tumors, 39.22% (40/102) of G2 tumors, and 57.41% (124/216) of G3 tumors. The clinical characteristics of these patients and tumors are shown in Table 1. The subset of 30 patients with specimens showing multiple pathologies were analyzed, to evaluate the heterogeneity of the Ki-67 index throughout the disease course (Table 2).

**Table 1:** Clinical characteristics in 514 patients with GEP-NENs

Variables	Total *n* = 514 (%)
Sex	
Male	302 (58.75)
Female	212 (41.25)
Grade (Ki-67)	
G1	196 (38.13)
G2	102 (19.84)
G3	216 (42.02)
Primary tumor site	
Pancreas	149 (28.99)
Large colon	148 (28.79)
Stomach	100 (19.46)
Esophagus	22 (4.28)
Duodenum	16 (3.11)
Other sites	79 (15.37)
Metastasis	182 (35.41)
Age at diagnosis (years)	55 (12–85)

**Table 2:** Ki-67 index variability and WHO class change in patients with multiple pathology specimens

Patient #	Primary tumor site Site1#	Metastatic/Re-biopsy tumor site Site2#	Ki-67 index (%)		Survival time	WHO class change
			Site1#	Site2#		
1	pancreas	peritoneum	70	70	12	–
2	pancreas	lymph node	10	30	15	G2 → G3
3	pancreas	peritoneum	5	20	6	G2 → G2
4	pancreas	liver	5	5	11	–
5	pancreas	liver	2	2	12	–
6	pancreas	pancreas	1	1	24	–
7	pancreas	liver	1	1	38	–
8	rectum	lymph node	70	70	12	–
9	rectum	liver	50	80	16	G3 → G3
10	rectum	liver	2	5	13	G1 → G2
11	rectum	liver	2	1	5	G1 → G1
12	rectum	liver	2	5	3	G1 → G2
13	rectum	lymph node	70	90	8	G3 → G3
14	rectum	lymph node	2	5	7	G1 → G2
15	rectum	liver	2	2	14	–
16	rectum	liver	10	10	–	–
17	rectum	rectum	80	60	12	G3 → G3
18	rectum	lymph node	60	80	–	G3 → G3
19	stomach	lymph node	2	5	12	G1 → G2
20	stomach	lymph node	60	80	14	G3 → G3
21	stomach	liver	1	1	24	–
22	stomach	lymph node	60	40	22	G3 → G3
23	stomach	lymph node	5	5	27	–
24	stomach	stomach	50	80	–	G3 → G3
25	stomach	stomach	70	30	33	G3 → G3
26	stomach	stomach	30	40	21	G3 → G3
27	stomach	pancreas	70	70	8	–
28	stomach	stomach	5	5	60	–
29	ileocecal junction	lymph node	70	70	16	–
30	duodenum	liver	5	5	12	–

### Variability of Ki-67 throughout the disease course of NENs

Among the 30 patients, 10 (33.3%) were G1; 7 (23.3%) G2; and 13 (43.3%) G3. Assessment of the Ki-67 index in those 30 cases with specimens showing multiple pathologies revealed discrepancies in 53.3% cases, among which, 40% and 13.3% patients had up-regulated and down-regulated Ki-67 levels, respectively. The up-regulation of the Ki-67 index from primary to metastatic specimens, or during the disease course was as follows: G1 to G2, 25.0% (4/16); G2 to G3, 6.25% (1/16); G2 to G2, 6.25% (1/16); G3 to G3, 37.5% (6/16).

**Fig. 1:**
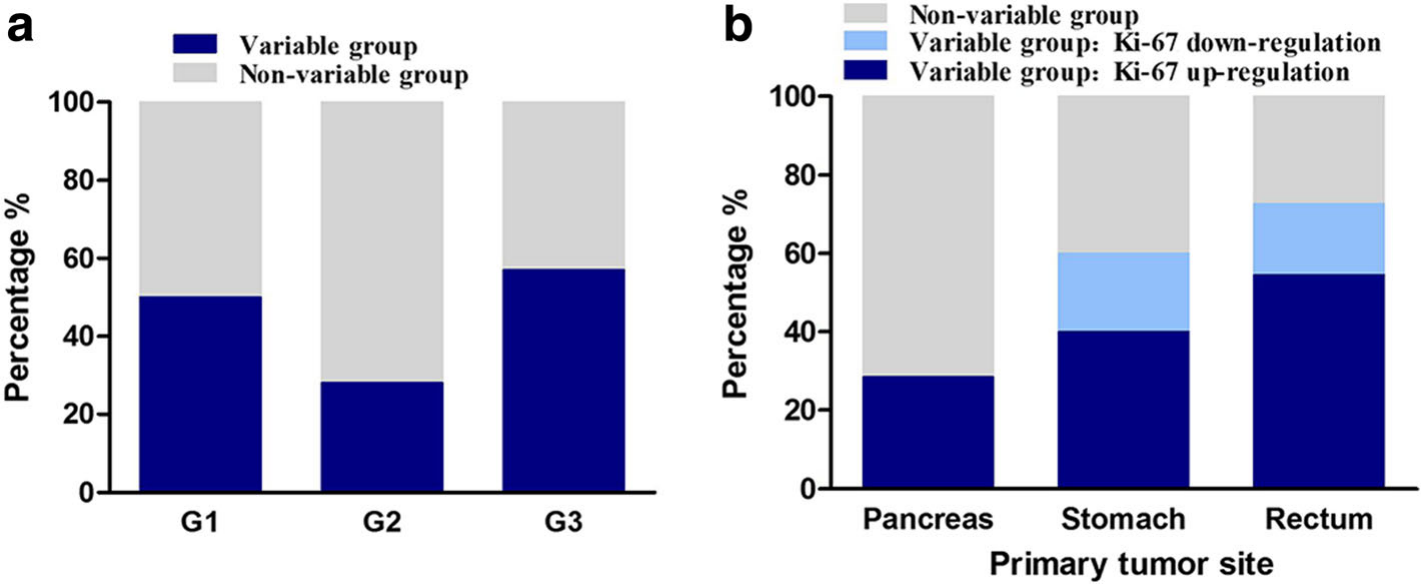
Distribution of variable cases according to GEP-NENs grade (**a**) and primary tumor site (**b**)

Some Ki-67 variability was observed in 41.18% (7/17) of the patients with primary tumors categorized as G1/G2; 35.29% (6/17) showed up-regulation of Ki-67 and 5.88% (1/17) showed down-regulation of Ki-67 (Fig. 1a). For primary tumors categorized as G3 (as confirmed by both Ki-67 and mitotic count), the Ki-67 variability was 57.1% (9/13), including 46.15% (6/13) showing up-regulation, and 23.08% (3/13) showing down-regulation (Fig. 1a).

The rectum was the most variable of the primary sites, whereas grade 3 (G3) tumors had the most variable intervals. Among the 11 patients with primary tumors in the rectum, Ki-67 variability was present in 72.72% (8/11) of all cases; 54.54% (6/11) showed up-regulation, and 18.18% (2/11) showed down-regulation (Fig. 1b). About 28.6% of the patients with primary tumors in the pancreas had up-regulated Ki-67 levels in metastatic loci and were upstaged to a higher WHO class (from G2 to G3) (Table 2, Fig. 1b). For ten patients with primary tumors in the stomach, 60% (6/10) showed Ki-67 variability, with 40% (4/10) showing up-regulation, and 20% (2/10) showing down-regulation of Ki-67 levels (Fig. 1b).

### Survival analysis

**Fig. 2:**
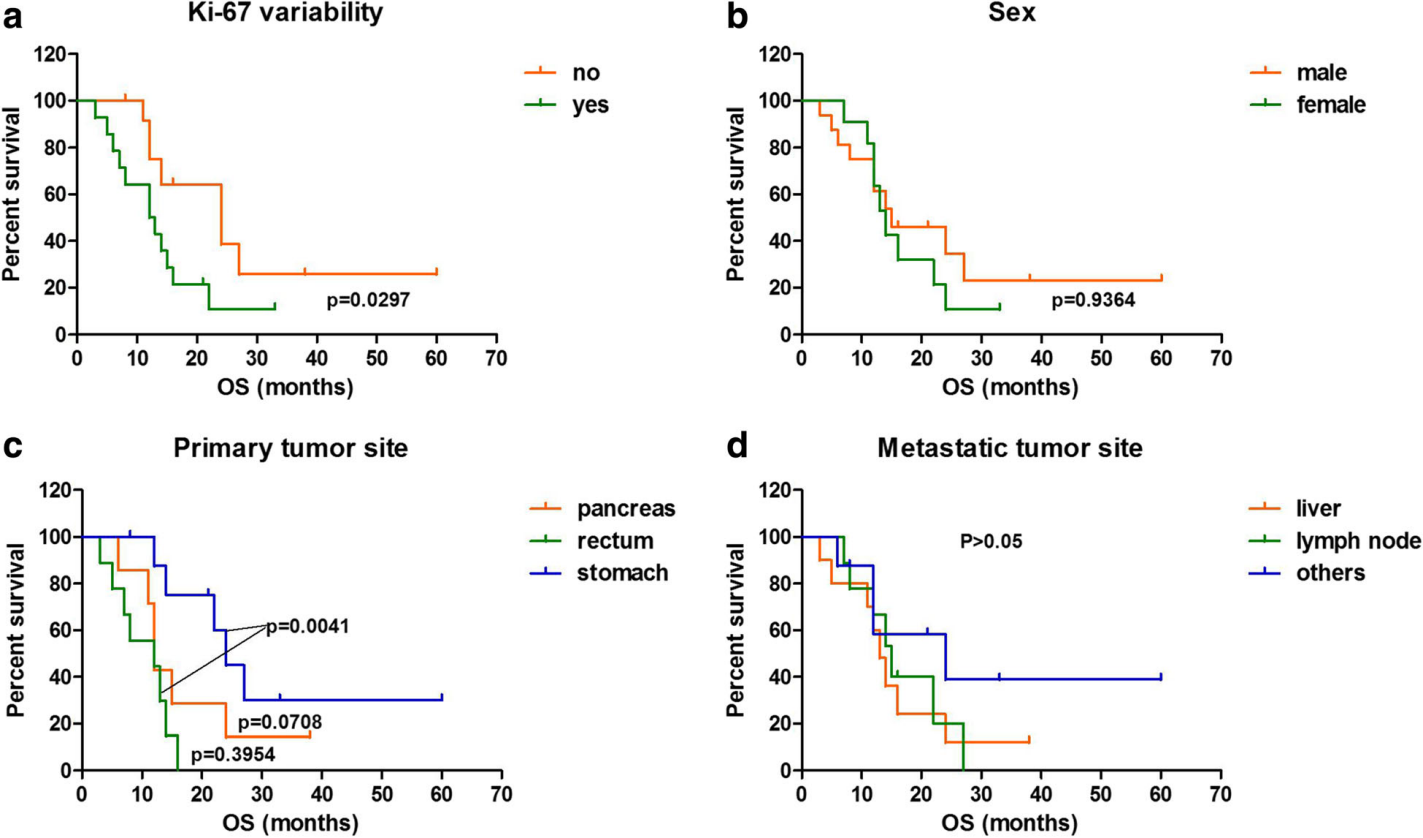
Survival curves in patients with GEP-NENs according to (**a**) Ki-67 index variability, (**b**) Sex, (**c**) Primary tumor site, (**d**) Metastatic tumor site

Kaplan–Meier survival analysis showed a significant discrepancy in mortality between Ki-67 variable and non-variable groups; the group with Ki-67 variability had a poorer prognosis than the group without Ki-67 variability (*p* = 0.0297) (Fig. 2). Cox regression analysis included age, sex, primary and metastatic tumor sites, Ki-67 level, and Ki-67 variability, and showed that Ki-67 variability (*p* = 0.038) was the only prognostic factor for survival in patients with metastatic GEP-NENs (Table 3).

**Table 3:** Cox regression analysis on prognostic baseline factors for survival in patients with multiple pathology specimens

Characteristics	Univariate analysis		Multivariate Cox regression analysis	
	HR (95%CI)	*P* value	HR (95%CI)	*P* value
Age	0.994 (0.954–1.037)	0.789		
Sex, female vs. Male	1.312 (0.530–3.248)	0.559		
Primary tumor site		0.068		0.310
Pancreas vs. Stomach	2.379 (0.720–7.852)	0.155	2.031 (0.564–7.311)	0.278
Rectum vs. Stomach	5.468 (1.570–19.041)	0.008	3.320 (0.956–11.523)	0.059
Metastasis, Yes vs. No	6.142 (0.810–46.551)	0.079	6.963 (0.814–59.588)	0.076
Ki-67 index	0.995 (0.979–1.011)	0.565		
Ki-67 Variability, Yes vs. No	2.612 (0.999–6.834)	0.045	3.487 (1.069–11.380)	0.038

## Discussion

In this study, we explored the relationship between the Ki-67 variability and the prognosis of patients with GEP-NENs. Our data support the fact that the Ki-67 index in GEP-NENs can change throughout the disease course, often with progression to increased malignancy and greater aggressiveness after metastasis. Moreover, this study further demonstrates that patients with Ki-67 variability have a poorer prognosis. Thus, re-assessment of Ki-67 at the sites of metastases, or during the disease course may prove to be a significant step in determining the prognosis of patients with GEP-NENs.

The GEP-NENs represent a heterogeneous family with variable biological and clinical characteristics [7, 12, 13]. Over the last few decades, neuroendocrine tumors (NETs) have been commonly considered rare tumors. However, the real incidence and prevalence of NETs, that has increased 6.4-fold from 1973 (1.09 per 100,000) to 2012 (6.98 per 100,000), according to Surveillance, Epidemiology, and End Results (SEER) data, may be underestimated [14].

Although the role of the Ki-67 index in GEP-NENs has been widely recognized since 2004, some recent studies have found that the Ki-67 index might change throughout the disease course or between primary and metastatic sites [1, 2, 11, 15]. One UK study showed that Ki-67 variability existed in 41.2% GEP-NEN cases from the primary tumor to metastatic sites [1]. Singh et al. (2014) proposed that Ki-67 might vary during the disease course, from primary stage to metastasis, and these changes throughout the course of the disease might have a significant impact on the monitoring and management of NETs [2]. Along with those observations, we also found that variability of the Ki-67 index between primary and metastatic specimens, or during the disease course was identified in about 53.3% of the patients in the present study.

We further analyzed the cases with Ki-67 index variability. Among 30 patients, the Ki-67 levels were found to be up- and down-regulated in 40% and 13.3% of the cases, respectively from the primary site to the metastatic site or during the disease course. The up-regulation of Ki-67 levels was as follows: G1 to G2, 25.0%; G2 to G3, 6.25%; G3 to G3, 37.5%. Shifting to a higher grade was mainly observed between G1 and G2, and as both G1 and G2 tumors received the same treatment, the clinical management based on the current criteria was not affected [16, 17]. In about 6.25% of the patients showing Ki-67 index variability, the tumor grades were upstaged from G2 to G3. However, without re-assessments of the metastasis or during the disease course, those G1/G2 NENs might not have been recognized as potential candidates for chemotherapy. Therefore, our results suggest that identification of the upstaged subset of G1/G2 was significantly useful to clinical doctors in determining appropriate treatment options and evaluating prognosis.

Although previous papers [1, 2, 11] have advocated re-assessment of the Ki-67 index throughout the disease course, or the progression from primary to metastatic sites, reports on whether variability in Ki-67 levels affects the prognosis of GEP-NENs are lacking. Kaplan–Meier survival analysis of the patients of the present study showed that the group with Ki-67 variability had a significantly shorter overall survival. Furthermore, results of the Cox regression analysis further confirmed that Ki-67 variability was the only independent prognostic factor for survival in those patients. These results emphasize the need for biopsies from metastatic lesions, or over the course of the disease. Moreover, the assessment of Ki-67 levels at all sites could significantly improve patient management.

To date, the reasons for Ki-67 index variability in GEP-NENs remain unclear. Miller et al. [1] hypothesized that variation of Ki-67 expression within a tumor is due to genetic intramural heterogeneity of NENs, as had been shown in other solid cancers [18–20]. However, Singh et al. [2] supposed that Ki-67 index changes during the disease course could be due to treatment effects and therapy resistance. Although the present study showed that the Ki-67 index varies from primary to metastatic sites, or during the disease course, we were not able to draw solid conclusions about the reasons behind the Ki-67 variability observed because of the relatively small sample size. The underlying mechanisms need to be explored in further studies with larger sample sizes.

## Conclusions

In summary, our study confirms that discordant expressions of Ki-67 in primary tumors and metastases are common in GEP-NENs. Furthermore, we also presented strong evidence that patients with Ki-67 variability have a poorer prognosis in GEP-NENs, and there is need for increased vigilance with this subgroup. Therefore, we recommend as a matter of great importance, a re-biopsy and re-estimation of the Ki-67 index at metastatic sites, or during the disease course. Although both intra-tumor heterogeneity and therapy resistance are speculated to be the underlying mechanisms of Ki-67 variability, the manner in which they affect patient prognosis requires further study.

## Abbreviations

GEP-NENs: Gastroenteropancreatic Neuroendocrine Neoplasms
NETs: Neuroendocrine tumors
OS: Overall survival
SEER: Surveillance, Epidemiology, and End Results program
WHO: World Health Organization
